# Discoloration after Regenerative Endodontic Procedures: A Critical Review

**DOI:** 10.22037/iej.v13i3.21271

**Published:** 2018

**Authors:** Radovan Žižka, Jiří Šedý, Ladislav Gregor, Iva Voborná

**Affiliations:** a *Czech Educational Dental Research Innovative Group, Brno, Czech Republic; *; b *Department of Anatomy, Faculty of Medicine and Dentistry, Palacky University, Olomouc, Czech Republic; *; c *Institute of Dentistry and Oral Sciences, Faculty of Medicine and Dentistry, Palacky University, Olomouc, Czech Republic*

**Keywords:** Biodentin, Calcium Hydroxide, Calcium Silicate, CEM cement, Mineral Trioxide Aggregate, Regenerative Medicine, Sodium Hypochlorite, Tooth Discoloration

## Abstract

Discoloration remains an unfavourable complication of otherwise successful regenerative endodontic procedure of immature teeth with necrotic pulp. This review presents a critical view on current knowledge of discoloration sources, its treatment and possible preventive modalities, dealing mainly with the use of antibiotics, ethylendiaminotetraacetic acid, calcium hydroxide, mineral trioxide aggregate, calcium silicate cements, sodium hypochlorite and chlorhexidin during regenerative treatment and their possible interactions. Bleaching as a discoloration treatment modality is discussed as well.

## Introduction

Regenerative endodontic procedure (REP) of immature teeth with necrotic pulp has become an important part of endodontic treatment modalities. It is considered as an alternative to calcium hydroxide apexification or mineral trioxide aggregate (MTA) apical plug [1]. REP is based on principles of tissue engineering in root canal treatment with the aim to regenerate pulp-dentinal organ [2]. Although the regeneration and production of dentin during REP is not completely understood yet [2], there is an evidence of possible continuing root development and thickening of the root canal wall [3, 4]. However, the predictability of this type of the treatment is still questionable [5]. These outcomes have been described as *scientist-based* or *clinician-based *[1]. There is increasing need to focus on outcomes that are meaningful to patients because of their increased participation in treatment decisions [1, 6]. *Patient-based* outcomes include resolving signs and symptoms of disease, survival of functional tooth in mouth and acceptable esthetics. Despite high success rate in terms of periodontal healing in comparison with apical plug with calcium silicate cement [7] the concerns about post-treatment tooth discoloration are increasing [8]. This outcome is very unfavorable in terms of patient´s smile esthetics despite otherwise excellent *clinician *and* science-based *outcomes. Thus, it can lead to the requirement to change treatment option.

The purpose of this article was to undertake a literature review of published studies on *i)* possible mechanisms of tooth discoloration after the use of triple antibiotic paste in REP, *ii)* possible mechanisms of tooth discoloration after the use of calcium silicate cements in REP, *iii)* potential preventive strategies precluding the tooth discoloration after REP, and *iv)* the effectiveness of internal bleaching as a strategy to solve tooth discoloration after REP.

***Triple antibiotic paste and discoloration***

The first cases of gray discoloration in REP were observed when the triple antibiotic paste (TAP) has been used, especially when minocycline was included [9, 10]. The ability of tetracycline to intrinsically stain teeth during odontogenesis is well-known for a long period of time [11]. The major factors affecting the amount of tetracycline deposition in sound tooth substances are its dosage, duration of treatment, stage of tooth mineralization and activity of the mineralization process [12]. The concentration of topically applied TAP can reach 1 g/mL which can be considered as a high concentration. Moreover, propylene glycol, which facilitate penetration of intracanal medication into dentin, is used as a vehicle [13]. The penetration can be improved if smear layer is removed [14]. TAP can be found as deep as 350 µm in dentin [15]. Despite the fact that TAP is recommended to stay in root canal not longer than 4 weeks, which can be considered as a short time exposure, the removal of paste is very limited [15, 16]. Moreover, some tissues have a high affinity for minocycline, especially tissues with high content of collagen such as dentin, bone, dental pulp, *etc*. [17]. The activity of mineralization process itself probably do not play a significant role in discoloration after REP because it is based on incorporation of intrinsic minocycline into tooth structure after its systemic administration [18]. 

There are several proposed mechanisms of minocycline staining [18]. As mentioned before, the incorporation of intrinsic minocycline from blood plasma is unfeasible. Another possible mechanism is its linkage to collagen molecules and its further oxidation and transformation to pigmented byproducts [17]. It has been demonstrated on animal models that oxidative reaction and subsequent pigment formation could be blocked by an antioxidant such as vitamin C [19]. We thus postulate that during the REP the antioxidant (such as vitamin C) should be included into the TAP. The last mechanism is based on unparalleled property of minocycline to chelate with iron and produce insoluble complexes [20]. Some calcium silicate cements could be a possible source of iron ions [21] as well as the blood clot used as a matrix. There are only limited possibilities how to avoid or even reduce the discoloration if TAP with minocycline is used. It has been proposed to adhesively seal the pulp chamber in order to obturate the dentin tubules and limit the contact of TAP with the dentin [10]. Although the application of dentin bonding agent do not completely prevent the clinically perceptible coronal color change, it has an ability to significantly reduce the tooth discoloration [22]. Another approach is to substitute minocycline with another antibiotic or to use double antibiotic paste where the minocycline is excluded [14, 23, 24]. However, even with substitution of minocycline with amoxicillin, clindamycin or cefaclor, the clinically significant staining potential still remains [25-29]. Although the use of double antibiotic paste leads to discoloration below the human eye perceptibility threshold [26], they do not guarantee color stability of teeth [30]. Other possibility is to use calcium hydroxide as an intracanal medication. The occurrence of discoloration in REP using calcium hydroxide is lower [8], its removal is much easier than TAP [15], it stimulates the release of the growth factors from dentinal matrix [31] and the condition of dentin with calcium hydroxide has positive effect on survival of stem cells of apical papilla (SCAP) [32]. In case the discoloration has already occurred, the internal bleaching of crown could be attempted. Despite the promising outcome of *in vitro* study of discolored crowns [25, 33], the reversion of discoloration in clinical cases is rather limited or exhibit only partial success [9, 27]. 

***Calcium silicate cements and discoloration***

Despite the decrease in the use of TAP and its substitution by calcium hydroxide, the level of occurrence of cervical discoloration after REP still remains. There are suggestions it may be caused by calcium silicate cements which are used as a coronal barrier in REP. The most employed calcium silicate cement in REP is ProRoot MTA (Dentsply-Maillefer, Ballaigues, Switzerland) or materials derived from MTA [34]; but in literature, the use of another calcium silicate cements such as Biodentin (Septodont, St. Maur-de- Fossés, France) [35-37], EndoSequence (Brasseler USA, Savannah, USA) [38] or calcium enriched mixture cement (CEM cement) (BioniqueDent, Tehran, Iran) [39-41] has been described. Most theories about the mechanism of tooth discoloration include oxidation of the heavy metal oxides (*i.e*. iron, bismuth or manganese) contained in calcium silicate cements [42]. Firstly, it was postulated to be caused by iron (alluminoferrite phase of powder) and manganese which were contained in original gray ProRoot MTA [21, 43]. This manifested as grayish staining was described in pulpotomies of primary molars [44], pulp capping [45, 46], apexogenesis [47], perforation management [48] or treatment of horizontal tooth fractures [49]. This unfavorable discoloration led to introduction of white MTA with modified formula without iron and manganese [21, 50]. The discoloration does not occur after setting of this material *in vitro*. Nevertheless, the staining of this new formula was obvious in *in vitro* experiments [51, 52]. Although the color change was greater with gray MTA, both of them induced clinically perceptible crown discoloration [53]. That focused the research on bismuth because the materials derived from ProRoot MTA which employed bismuth as a radiopacifier have higher discoloration potential than materials which use different radiopacifier [42] . It was found that bismuth is bound to calcium silicate hydrate and leached from material as the calcium silicate hydrate decomposed with time [54]. The content of bismuth oxide in powder of ProRoot MTA is 21.6% and after setting the content in material is only 8.4% [54]. 

There are several mechanisms how bismuth can cause discoloration. The first and in REP the most important, is the interaction of bismuth oxide with collagen which is converted into black precipitate [55]. In REP the prolonged irrigation with EDTA to expose collagen matrix with embedded growth factors remains a part of the treatment protocol. The second possible mechanism is an exposure of bismuth oxide to higher temperatures and light irradiation in oxygen-free environment [56-58]. It was shown that greater duration of light curing lead to color change in CEM cement and white MTA, but the color stability was lower if white MTA was used [59]. While setting of calcium silicate cements occurs in oxygen-free environment, the light irradiation is limited to curing of adhesive reconstruction and temperature does not exceed body temperature. The last-mentioned mechanism is the reaction of oxidized bismuth oxide with carbon dioxide which leads to production of bismuth carbon which causes discoloration [57, 60]. This particular mechanism is not likely to happen in REP because the material sets in root canal in the absence of air. Moreover, the binding of carbon dioxide during blood clot setting inside the tooth might be possible mechanism protecting against bismuth carbon production; however, no scientific data on the levels of carbon dioxide inside blood clot during its setting are available yet, so this remains a hypothesis which needs to be confirmed or excluded in future studies. It is necessary to point out that discoloration manifests gradually during several weeks and months because the bismuth is released from calcium silicate hydrate successively [54]. Materials with content of bismuth are supposed to be materials with higher discoloration potential [42, 52, 61-63], however some studies observed opposite outcomes [64].

Other possible causes are reactions of calcium silicate cements with irrigation solutions which are used during REP. The most utilized disinfection solution in REP is sodium hypochlorite. It has been described that some calcium silicate cements did not change the color if they set in the contact with sodium hypochlorite [65]. It was observed that bismuth oxide, in contact with dental structure immersed in sodium hypochlorite, exhibited a change in color from light yellow to dark brown [55]. The calcium silicate cements without bismuth do not change the color when set in contact with sodium hypochlorite [66] or the discoloration is less noticeable [67]. Despite the fact that sodium hypochlorite is vastly used in REP [34, 68], it is not very probable that sodium hypochlorite will be present at the time of application of calcium silicate cement. One of the major steps in *Clinical Consideration for Regenerative Procedures* by *American Association of Endodontists* is prolonged irrigation with EDTA [69]. It was proved that EDTA interacts with sodium hypochlorite in aqueous solutions and grossly decrease free chlorine and tissue-dissolving capacity of sodium hypochlorite [70]. Therefore, this mechanism of discoloration during REP is improbable if these instructions are followed. However, it was showed that in 70% of published studies the final irrigation used was sodium hypochlorite [34]. On the other hand, the presence of sodium hypochlorite can play a significant role in development of discoloration in vital pulp therapy where the highly concentrated hypochlorite has been used [71].

**Table 1:** Discoloration of calcium silicate cements used in REP

**Material**	**Radiopacifier**	**Discoloration after conctact**					
		**Setting**	**Dentin**	**Sodium Hypochlorite **	**Chlorhexidin **	**EDTA **	**Blood **
**ProRoot MTA gray**	**Bismuth oxide**	+	++	++	+	+	+
**ProRoot MTA white**	**Bismuth oxide**	-	++	++	+	+	+
**Biodentin**	**Zirconium oxide**	-	-	+	+	-	+
**Endosequence BC RRM**	**Tantalum pentoxide**	-	-	+	+	-	+
**CEM cement**	**Barium sulfate**	-	-	+	+	-	+

**Table 2.** Mechanisms of discoloration in REP

**Material**	**Component/reaction**	**Mechanism of discoloration**
**TAP**	**Minocyclin**	Bound to collagen and oxidation [17]
		Chelation with iron [20]
**Calcium silicate cement**	**Content of Fe, Mn [21]**	Free d-electrons with oxidation
	**Bismuth**	Precipitation with collagen [55]
		Light irradition in oxygen-free [58]
		Reaction with sodium hypochlorite [55, 65]
		Exposure to high temperatures [58]
		Reaction with EDTA [66]
	**Reaction with chlorhexidin [67]**	
	**Reaction with blood [76]**	

Regardless the current recommendation of AAE, limited amount of studies used chlorhexidine in REP [34]. It is well known that chlorhexidine cause tooth surface discoloration [72]. Also, chlorhexidine has significant affinity to dentin and relevant amount of chlorhexidine remain in dentin after exposure [73]. In REP, the chlorhexidine should be avoided, because of its detrimental effect on survival of SCAP which cannot be reversed by another irrigation solution [74]. Beside this adverse effect it causes discoloration of all calcium silicate cements [66, 67]. Therefore, the use of chlorhexidine should be avoided not only from biological point of view but from esthetic reasons as well. Another recommended irrigation solution in REP is EDTA. There is only limited evidence on discoloration potential of EDTA with calcium silicate cements. The mechanism is not known but in one study only material with bismuth oxide had significantly higher discoloration when exposed to EDTA during setting [66]. Because EDTA is the final irrigation solution which used in REP, it is most likely that some amount of EDTA remains in root canal system. Therefore, if future research confirms the discoloration potential of EDTA on some calcium silicate cements, it would be feasible to rinse root canal system with saline or use calcium silicate cement without bismuth oxide.

Another possible cause of discoloration of calcium silicate cement is its contact with blood [43]. Blood by itself could also lead to discoloration by the accumulation of hemoglobin, its metabolites or other forms of hematin molecules in dentin [75]. Staining of calcium silicate cements is observable when material sets in contact with red blood cells [76], so we can assume that iron ions and development of calcium allumino ferrate can be relevant in the origin of discoloration. Another possible explanation is the presence of porosities in calcium silicate cement which can absorb blood components [77]. In view of the fact that blood clot is the most spread matrix used in REP [34], there a great emphasis should be put on this possible source of discoloration. High affinity to discoloration in contact with blood is expressed in all calcium silicate cements [43], even with the calcium silicate cements which are supposed to have lower discoloration potential [42]. The possible prevention of discoloration is the use of autogenous matrix without red blood cells such plasma rich on platelets (PRP) or the use of synthetic matrix. Another alternative is to mix calcium silicate cement with propylene glycol which leads to reduction of staining [78]. The ideal concentration of propylene glycol is regarded to be 20% of propylene glycol in distilled water [79]. Moreover, it is feasible to place the coronal barrier more apically, so the discoloration of tooth structure will be covered by bone or gingiva. The information about calcium silicate cements and discoloration are summarized in Table 1. We must highlight that available evidence about the use of other calcium silicate cements than ProRoot MTA in REP is limited. The possible etiopathogenetic factors leading to discoloration, its prevention and therapy are summarized in Table 2.

***Bleaching as a treatment of discolorations in REP***

Despite the growing evidence about occurrence of discoloration in REP, there is only scarce evidence about correction of discoloration. Majority of information we have, are about internal bleaching of discolorations caused by TAP. Kirchhoff *et al*. [33], in an *in vitro* study on extracted teeth reported that teeth treated with TAP with minocycline can be bleached successfully with sodium perborate paste. When the bleaching potential of sodium perborate and 35% hydrogen peroxide were compared, it has been found that bleaching with 35% hydrogen peroxide is more effective [25]. This is in agreement with study by Lim *et al*. [80] who found 35% hydrogen peroxide to be more effective than sodium perborate for intracoronal bleaching of blood discolored crowns. When the carbamide peroxide is used, the bleaching of discolored tooth crown has only partial effect [81]. Despite these *in vitro* outcomes, the bleaching in clinical setting is rather unsatisfactory [9, 27, 81]. This may be cause by the persistence of minocycline deeply inside the dentinal tubules [15] or by the remaining presence of calcium silicate coronal barrier, which has not been removed completely [9]. The bleaching agent cannot reach the discoloration under the coronal barrier or deep in the dentin. Moreover, the agent is not able to bleach discolored calcium silicate cement. Jang *et al.* [82] presented *in vitro* study where the removal of discolored calcium silicate cement contributed more to resolving tooth discoloration than internal bleaching. So, it is highly recommended to remove the discolored coronal barrier before placing the bleaching agent. This technique has been described in successful case reports [46, 83]. Since we do not have proper knowledge about the success rate and longevity of internal bleaching of discolored teeth treated with REP, the main emphasis should be put on prevention of discoloration origin and reduction of its occurrence.

## Conclusion

The discoloration of teeth treated with REP, caused by antibiotic paste can be prevented only partially. The use of calcium hydroxide as an intracanal medication agent should be preferred. When calcium silicate cements are used for coronal barrier, the priority should be given to calcium silicate cements without bismuth which have lower discoloration potential. Nevertheless, even these cements can be discolored in contact with the blood. The discoloration of calcium silicate cements with PRP or synthetic matrixes should be further investigated. Besides that, the focus should be put on possibility of the application of calcium silicate coronal barrier more apically. If the discoloration is anticipated and recommendations are meticulously followed, the level of discoloration of teeth can be minimized below the human eye threshold.

## References

[1] Diogenes A, Ruparel NB, Shiloah Y, Hargreaves KM (2016). Regenerative endodontics: A way forward. J Am Dent Assoc.

[2] Zizka R, Sedy J (2017). Paradigm Shift from Stem Cells to Cell-Free Regenerative Endodontic Procedures: A Critical Review. Stem Cells Dev.

[3] Kahler B, Rossi-Fedele G, Chugal N, Lin LM (2017). An Evidence-based Review of the Efficacy of Treatment Approaches for Immature Permanent Teeth with Pulp Necrosis. J Endod.

[4] Chen YP, Jovani-Sancho Mdel M, Sheth CC (2015). Is revascularization of immature permanent teeth an effective and reproducible technique?. Dent Traumatol.

[5] Nazzal H, Duggal MS (2017). Regenerative endodontics: a true paradigm shift or a bandwagon about to be derailed?. Eur Arch Paediatr Dent.

[6] Barry MJ, Edgman-Levitan S (2012). Shared decision making--pinnacle of patient-centered care. N Engl J Med.

[7] Torabinejad M, Nosrat A, Verma P, Udochukwu O (2017). Regenerative Endodontic Treatment or Mineral Trioxide Aggregate Apical Plug in Teeth with Necrotic Pulps and Open Apices: A Systematic Review and Meta-analysis. J Endod.

[8] Kahler B, Rossi-Fedele G (2016). A Review of Tooth Discoloration after Regenerative Endodontic Therapy. J Endod.

[9] Kim JH, Kim Y, Shin SJ, Park JW, Jung IY (2010). Tooth discoloration of immature permanent incisor associated with triple antibiotic therapy: a case report. J Endod.

[10] Reynolds K, Johnson JD, Cohenca N (2009). Pulp revascularization of necrotic bilateral bicuspids using a modified novel technique to eliminate potential coronal discolouration: a case report. Int Endod J.

[11] Wallman IS, Hilton HB (1962). Teeth pigmented by tetracycline. Lancet.

[12] Cohlan SQ (1977). Tetracycline staining of teeth. Teratology.

[13] Cruz EV, Kota K, Huque J, Iwaku M, Hoshino E (2002). Penetration of propylene glycol into dentine. Int Endod J.

[14] Sato I, Ando-Kurihara N, Kota K, Iwaku M, Hoshino E (1996). Sterilization of infected root-canal dentine by topical application of a mixture of ciprofloxacin, metronidazole and minocycline in situ. Int Endod J.

[15] Berkhoff JA, Chen PB, Teixeira FB, Diogenes A (2014). Evaluation of triple antibiotic paste removal by different irrigation procedures. J Endod.

[16] Arslan H, Capar ID, Saygili G, Uysal B, Gok T, Ertas H, Topcuoglu HS (2014). Efficacy of various irrigation protocols on the removal of triple antibiotic paste. Int Endod J.

[17] Bowles WH, Bokmeyer TJ (1997). Staining of adult teeth by minocycline: binding of minocycline by specific proteins. J Esthet Dent.

[18] Sanchez AR, Rogers RS 3rd, Sheridan PJ (2004). Tetracycline and other tetracycline-derivative staining of the teeth and oral cavity. Int J Dermatol.

[19] Bowles WH (1998). Protection against minocycline pigment formation by ascorbic acid (vitamin C). J Esthet Dent.

[20] Primosch RE (1980). Tetracycline discoloration, enamel defects, and dental caries in patients with cystic fibrosis. Oral Surg Oral Med Oral Pathol.

[21] Asgary S, Parirokh M, Eghbal MJ, Brink F (2005). Chemical differences between white and gray mineral trioxide aggregate. J Endod.

[22] Shokouhinejad N, Khoshkhounejad M, Alikhasi M, Bagheri P, Camilleri J (2017). Prevention of coronal discoloration induced by regenerative endodontic treatment in an ex vivo model. Clin Oral Investig.

[23] Thibodeau B, Trope M (2007). Pulp revascularization of a necrotic infected immature permanent tooth: case report and review of the literature. Pediatr Dent.

[24] Thomson A, Kahler B (2010). Regenerative endodontics--biologically-based treatment for immature permanent teeth: a case report and review of the literature. Aust Dent J.

[25] Yasa B, Arslan H, Akcay M, Kavrik F, Hatirli H, Ozkan B (2015). Comparison of bleaching efficacy of two bleaching agents on teeth discoloured by different antibiotic combinations used in revascularization. Clin Oral Investig.

[26] Akcay M, Arslan H, Yasa B, Kavrik F, Yasa E (2014). Spectrophotometric analysis of crown discoloration induced by various antibiotic pastes used in revascularization. J Endod.

[27] McTigue DJ, Subramanian K, Kumar A (2013). Case series: management of immature permanent teeth with pulpal necrosis: a case series. Pediatr Dent.

[28] Kahler B, Mistry S, Moule A, Ringsmuth AK, Case P, Thomson A, Holcombe T (2014). Revascularization outcomes: a prospective analysis of 16 consecutive cases. J Endod.

[29] Dabbagh B, Alvaro E, Vu DD, Rizkallah J, Schwartz S (2012). Clinical complications in the revascularization of immature necrotic permanent teeth. Pediatr Dent.

[30] Dettwiler CA, Walter M, Zaugg LK, Lenherr P, Weiger R, Krastl G (2016). In vitro assessment of the tooth staining potential of endodontic materials in a bovine tooth model. Dent Traumatol.

[31] Graham L, Cooper PR, Cassidy N, Nor JE, Sloan AJ, Smith AJ (2006). The effect of calcium hydroxide on solubilisation of bio-active dentine matrix components. Biomaterials.

[32] Althumairy RI, Teixeira FB, Diogenes A (2014). Effect of dentin conditioning with intracanal medicaments on survival of stem cells of apical papilla. J Endod.

[33] Kirchhoff AL, Raldi DP, Salles AC, Cunha RS, Mello I (2015). Tooth discolouration and internal bleaching after the use of triple antibiotic paste. Int Endod J.

[34] Kontakiotis EG, Filippatos CG, Tzanetakis GN, Agrafioti A (2015). Regenerative endodontic therapy: a data analysis of clinical protocols. J Endod.

[35] Chaniotis A (2017). Treatment Options for Failing Regenerative Endodontic Procedures: Report of 3 Cases. J Endod.

[36] Aldakak MM, Capar ID, Rekab MS, Abboud S (2016). Single-Visit Pulp Revascularization of a Nonvital Immature Permanent Tooth Using Biodentine. Iran Endod J.

[37] Bakhtiar H, Esmaeili S, Fakhr Tabatabayi S, Ellini MR, Nekoofar MH, Dummer PM (2017). Second-generation Platelet Concentrate (Platelet-rich Fibrin) as a Scaffold in Regenerative Endodontics: A Case Series. J Endod.

[38] Bukhari S, Kohli MR, Setzer F, Karabucak B (2016). Outcome of Revascularization Procedure: A Retrospective Case Series. J Endod.

[39] Nosrat A, Seifi A, Asgary S (2011). Regenerative endodontic treatment (revascularization) for necrotic immature permanent molars: a review and report of two cases with a new biomaterial. J Endod.

[40] Asgary S, Fazlyab M, Nosrat A (2016). Regenerative Endodontic Treatment versus Apical Plug in Immature Teeth: Three-Year Follow-Up. J Clin Pediatr Dent.

[41] Roghanizadeh L, Fazlyab M (2018). Revascularization and Apical Plug in an Immature Molar. Iran Endod J.

[42] Mozynska J, Metlerski M, Lipski M, Nowicka A (2017). Tooth Discoloration Induced by Different Calcium Silicate-based Cements: A Systematic Review of In Vitro Studies. J Endod.

[43] Shokouhinejad N, Nekoofar MH, Pirmoazen S, Shamshiri AR, Dummer PM (2016). Evaluation and Comparison of Occurrence of Tooth Discoloration after the Application of Various Calcium Silicate-based Cements: An Ex Vivo Study. J Endod.

[44] Maroto M, Barberia E, Planells P, Garcia Godoy F (2005). Dentin bridge formation after mineral trioxide aggregate (MTA) pulpotomies in primary teeth. Am J Dent.

[45] Glickman GN, Koch KA (2000). 21st-century endodontics. J Am Dent Assoc.

[46] Belobrov I, Parashos P (2011). Treatment of tooth discoloration after the use of white mineral trioxide aggregate. J Endod.

[47] Kvinnsland SR, Bardsen A, Fristad I (2010). Apexogenesis after initial root canal treatment of an immature maxillary incisor - a case report. Int Endod J.

[48] Bortoluzzi EA, Araujo GS, Guerreiro Tanomaru JM, Tanomaru-Filho M (2007). Marginal gingiva discoloration by gray MTA: a case report. J Endod.

[49] Yildirim T, Gencoglu N (2009). Use of mineral trioxide aggregate in the treatment of horizontal root fractures with a 5-year follow-up: report of a case. J Endod.

[50] Dammaschke T, Gerth HU, Zuchner H, Schafer E (2005). Chemical and physical surface and bulk material characterization of white ProRoot MTA and two Portland cements. Dent Mater.

[51] Boutsioukis C, Noula G, Lambrianidis T (2008). Ex vivo study of the efficiency of two techniques for the removal of mineral trioxide aggregate used as a root canal filling material. J Endod.

[52] Kohli MR, Yamaguchi M, Setzer FC, Karabucak B (2015). Spectrophotometric Analysis of Coronal Tooth Discoloration Induced by Various Bioceramic Cements and Other Endodontic Materials. J Endod.

[53] Ioannidis K, Mistakidis I, Beltes P, Karagiannis V (2013). Spectrophotometric analysis of coronal discolouration induced by grey and white MTA. Int Endod J.

[54] Camilleri J (2008). Characterization of hydration products of mineral trioxide aggregate. Int Endod J.

[55] Marciano MA, Duarte MA, Camilleri J (2015). Dental discoloration caused by bismuth oxide in MTA in the presence of sodium hypochlorite. Clin Oral Investig.

[56] Alsubait S, Al-Haidar S, Al-Sharyan N (2017). A Comparison of the Discoloration Potential for EndoSequence Bioceramic Root Repair Material Fast Set Putty and ProRoot MTA in Human Teeth: An In Vitro Study. J Esthet Restor Dent.

[57] Valles M, Roig M, Duran-Sindreu F, Martinez S, Mercade M (2015). Color Stability of Teeth Restored with Biodentine: A 6-month In Vitro Study. J Endod.

[58] Sanz O, Haro-Poniatowski E, Gonzalo J, Fernández Navarro JM (2006). Influence of the melting conditions of heavy metal oxide glasses containing bismuth oxide on their optical absorption. J Non Crystal Solids.

[59] Eghbal MJ, Torabzadeh H, Bagheban AA, Shamszadeh S, Marvasti LA, Asgary S (2016). Color stability of mineral trioxide aggregate and calcium enriched mixture cement. J Investig Clin Dent.

[60] Kang SH, Shin YS, Lee HS, Kim SO, Shin Y, Jung IY, Song JS (2015). Color changes of teeth after treatment with various mineral trioxide aggregate-based materials: an ex vivo study. J Endod.

[61] Marconyak LJ Jr, Kirkpatrick TC, Roberts HW, Roberts MD, Aparicio A, Himel VT, Sabey KA (2016). A Comparison of Coronal Tooth Discoloration Elicited by Various Endodontic Reparative Materials. J Endod.

[62] Ramos JC, Palma PJ, Nascimento R, Caramelo F, Messias A, Vinagre A, Santos JM (2016). 1-year In Vitro Evaluation of Tooth Discoloration Induced by 2 Calcium Silicate-based Cements. J Endod.

[63] Yoldas SE, Bani M, Atabek D, Bodur H (2016). Comparison of the Potential Discoloration Effect of Bioaggregate, Biodentine, and White Mineral Trioxide Aggregate on Bovine Teeth: In Vitro Research. J Endod.

[64] Beatty H, Svec T (2015). Quantifying Coronal Tooth Discoloration Caused by Biodentine and EndoSequence Root Repair Material. J Endod.

[65] Camilleri J (2014). Color stability of white mineral trioxide aggregate in contact with hypochlorite solution. J Endod.

[66] Sobhnamayan F, Adl A, Ghanbaran S (2017). Effect of Different Irrigation Solutions on the Colour Stability of Three Calcium Silicate-Based Materials. J Dent Biomater.

[67] Keskin C, Demiryurek EO, Ozyurek T (2015). Color stabilities of calcium silicate-based materials in contact with different irrigation solutions. J Endod.

[68] Diogenes A, Henry MA, Teixeira FB, Hargreaves KM (2013). An update on clinical regenerative endodontics. Endodontic Topics.

[69] Endodontics AAo Clinical considerations for regenerative procedures.

[70] Grawehr M, Sener B, Waltimo T, Zehnder M (2003). Interactions of ethylenediamine tetraacetic acid with sodium hypochlorite in aqueous solutions. Int Endod J.

[71] Bogen G, Kim JS, Bakland LK (2008). Direct pulp capping with mineral trioxide aggregate: an observational study. J Am Dent Assoc.

[72] Slot DE, Berchier CE, Addy M, Van der Velden U, Van der Weijden GA (2014). The efficacy of chlorhexidine dentifrice or gel on plaque, clinical parameters of gingival inflammation and tooth discoloration: a systematic review. Int J Dent Hyg.

[73] Carrilho MR, Carvalho RM, Sousa EN, Nicolau J, Breschi L, Mazzoni A, Tjaderhane L, Tay FR, Agee K, Pashley DH (2010). Substantivity of chlorhexidine to human dentin. Dent Mater.

[74] Trevino EG, Patwardhan AN, Henry MA, Perry G, Dybdal-Hargreaves N, Hargreaves KM, Diogenes A (2011). Effect of irrigants on the survival of human stem cells of the apical papilla in a platelet-rich plasma scaffold in human root tips. J Endod.

[75] Marin PD, Bartold PM, Heithersay GS (1997). Tooth discoloration by blood: an in vitro histochemical study. Endod Dent Traumatol.

[76] Felman D, Parashos P (2013). Coronal tooth discoloration and white mineral trioxide aggregate. J Endod.

[77] Lenherr P, Allgayer N, Weiger R, Filippi A, Attin T, Krastl G (2012). Tooth discoloration induced by endodontic materials: a laboratory study. Int Endod J.

[78] Guimaraes BM, Tartari T, Marciano MA, Vivan RR, Mondeli RF, Camilleri J, Duarte MA (2015). Color stability, radiopacity, and chemical characteristics of white mineral trioxide aggregate associated with 2 different vehicles in contact with blood. J Endod.

[79] Duarte MA, Alves de Aguiar K, Zeferino MA, Vivan RR, Ordinola-Zapata R, Tanomaru-Filho M, Weckwerth PH, Kuga MC (2012). Evaluation of the propylene glycol association on some physical and chemical properties of mineral trioxide aggregate. Int Endod J.

[80] Lim MY, Lum SO, Poh RS, Lee GP, Lim KC (2004). An in vitro comparison of the bleaching efficacy of 35% carbamide peroxide with established intracoronal bleaching agents. Int Endod J.

[81] Santos LG, Felippe WT, Souza BD, Konrath AC, Cordeiro MM, Felippe MC (2017). Crown discoloration promoted by materials used in regenerative endodontic procedures and effect of dental bleaching: spectrophotometric analysis. J Appl Oral Sci.

[82] Jang JH, Kang M, Ahn S, Kim S, Kim W, Kim Y, Kim E (2013). Tooth discoloration after the use of new pozzolan cement (Endocem) and mineral trioxide aggregate and the effects of internal bleaching. J Endod.

[83] Timmerman A, Parashos P (2017). Bleaching of a Discolored Tooth with Retrieval of Remnants after Successful Regenerative Endodontics. J Endod.

